# Canine β-defensin-1 (*CBD1*) gene as a possible marker for *Leishmania infantum* infection in dogs

**DOI:** 10.1186/s13071-017-2130-8

**Published:** 2017-04-20

**Authors:** Lidiane Gomes da Silva, César Raimundo Lima Costa-Júnior, Carlos Alberto Santiago Figueiredo-Júnior, Tereza Cristina Leal-Balbino, Sergio Crovella, Domenico Otranto, Valdir de Queiroz Balbino, Filipe Dantas-Torres

**Affiliations:** 1 Department of Genetics, Federal University of Pernambuco, Recife, Brazil; 2 Department of Microbiology, Aggeu Magalhães Institute, Oswaldo Cruz Foundation (Fiocruz), Recife, Brazil; 3 Department of Veterinary Medicine, University of Bari, Valenzano, Italy; 4 Department of Immunology, Aggeu Magalhães Institute, Oswaldo Cruz Foundation (Fiocruz), Recife, Brazil

**Keywords:** Canine β-defensin-1 gene, Single nucleotide polymorphisms, Canine leishmaniasis, Dogs

## Abstract

**Background:**

Canine leishmaniasis caused by *Leishmania infantum* is a parasitic disease of great veterinary significance. Some dogs infected by *L. infantum* may mount a strong cellular immune response and clear the infection, while others may respond with exaggerated antibody production against the parasite and develop an overt disease, which may be fatal, if left untreated. The initial factors triggering the polarization of the immune response towards a predominantly T-helper 1 or T-helper 2 cytokines, as well as the markers of resistance and susceptibility to *L. infantum* infection and disease development in dogs, are not fully understood. Herein, we assessed the association between single nucleotide polymorphisms (SNPs) in the canine β-defensin-1 (*CBD1*) gene and the infection by *L. infantum* in two dog populations from Brazil (Sobral in Ceará State and São Raimundo Nonato in Piauí State) and one dog population from Italy.

**Results:**

A total of 387 dogs were assessed for *L. infantum* by real time PCR and 34.6% of them were positive. In *CBD1* gene sequences from these positive dogs, nine polymorphic sites were detected, but only SNPs 3, 4, 7 and 8 were associated with *L. infantum*, in dogs from southern Italy. No association was found with dogs from Brazil*.*

**Conclusion:**

This study sets the basis for further studies on the usefulness of *CBD1* as a marker of *L. infantum* infection susceptibility in dogs.

**Electronic supplementary material:**

The online version of this article (doi:10.1186/s13071-017-2130-8) contains supplementary material, which is available to authorized users.

## Background

Canine leishmaniasis (CanL) is a disease caused by sand fly-transmitted protozoa of the genus *Leishmania*, which affects dogs in all continents, except Oceania [1]. From a global perspective, the most common causative agent of the disease is *Leishmania infantum*, the agent of zoonotic visceral leishmaniasis in humans. CanL caused by *L. infantum* is a widespread, life-threatening disease characterized by several, usually non-specific, clinical signs, such as weight loss, enlargement of lymph nodes, spleen and liver, lethargy, cutaneous and ocular lesions [2]. However, most of the *L. infantum*-infected dogs present neither evident clinical signs nor clinico-pathological abnormalities, being generally resistant to the infection [1, 2].

The variable clinical presentation of CanL depends on several predisposing factors, including breed, age and genetic background [2]. The development of overt disease is also dependent on the predominant type of immune response mounted by the dog against the parasites. It is known that a dominant T-helper 1 (Th1) cell-mediated immune response may lead to the resolution of infection, whereas an exaggerated T-helper 2 (Th2) cell proliferation can lead to a high antibody production, with the formation of immune complexes, which lead to clinical signs such as vasculitis, arthritis, uveitis, and glomerulonephritis [2, 3]. Undoubtedly, still many gaps remain about the initial factors triggering the polarization of the immune response as well as regarding the susceptibility and resistance markers for *L. infantum* infection in dogs.

Several studies have assessed the usefulness of immunological markers of susceptibility and resistance to *L. infantum* infection in dogs. For example, interleukin 10 (IL-10), tumour growth factor beta (TGF-β) and interleukin 4 (IL-4) are associated with increased B-cell and plasma-cell activity and hyperglobulinemia, and with disease susceptibility in dogs [3]. On the other hand, increased levels of interferon gamma (IFN-γ), interleukin 2 (IL-2) and tumour necrosis factor alpha (TNF-α) induce macrophage activation and nitric oxide intracellular killing of parasites, which is associated to resistance in naturally infected dogs [3–6]. Thus, the profile of the host immune response has been used as a marker of susceptibility or resistance to *L. infantum* infection in dogs.

It has been suggested that host genetics play a role in the susceptibility to CanL. For instance, the solute carrier family 11 member 1 gene (*SLC11A1*), formerly named resistance-associated macrophage protein 1 (*NRAMP1*) gene, and certain alleles of the major histocompatibility complex II (MHC II) genes have been associated with susceptibility to CanL [7, 8]. In a recent study, ~170,000 single nucleotide polymorphisms (SNPs) were genotyped in dogs and associations between *L. infantum* infection and polymorphisms were found in several genes (e.g. *Il7R*, *Lifr*, *C6, C7* and *Csf1r* genes), within a locus involved in lesion development in murine *Leishmania major* infection [9].

The association between SNPs and *L. infantum* infection or disease development in dogs is not so clear, since leishmaniasis is a complex disease whose progression is associated with multiple gene loci [10]. SNP analysis of innate immunity genes, such as those coding for antimicrobial peptides (AMPs), could provide interesting insights into the relationship between host’s genetics and *L. infantum* infection in dogs. Herein, we used molecular tools to determine the infection by *L. infantum* in dogs, living in three areas where CanL is endemic, and investigated the possible relationship between polymorphisms in the canine β-defensin-1 (*CBD1*) gene and positivity to *L. infantum*, as previous studies have demonstrated the association of polymorphisms in this gene and diseases in dogs [11, 12].

## Methods

### Dog populations and samples

A total of 387 mongrel dogs were included in this study, of which 95 from São Raimundo Nonato (09°00'54"S, 42°41'56"W), Piauí State, and 190 from Sobral (3°41'15"S, 40°21'5"W), Ceará State, north-eastern Brazil. Additionally, 102 dogs from Putignano (40°51'N, 17°7'E), Apulia region in southern Italy, were also included. Samples from Brazil were primarily collected for this study. The samples from Italy came from a previous study [13]. The selected populations live in known endemic areas of CanL. Venous blood samples (5 ml) from all dogs were collected in tubes containing anticoagulant and stored at 4 °C until DNA extraction.

### Molecular procedures

#### DNA extraction

DNA extraction from whole blood samples was performed using the salting-out method [14], for the Brazilian samples, and a commercial kit (MagAttract DNA Blood kit, Qiagen, Valencia, California, USA), for the Italian samples. Each extracted DNA sample was quantified by NanoDrop^TM^ 2000 (Thermo Scientific, Wilmington, Delaware, USA) to determine its concentration and purity.

#### Leishmania DNA detection

For *L. infantum* detection, the primers LEISH-1 (5'-AAC TTT TCT GGT CCT CCG GGT AG-3'), LEISH-2 (5'-ACC CCC AGT TTC CCG CC-3') and the TaqMan® probe FAM-5'-AAA AAT GGG TGC AGA AAT-3'-non-fluorescent quencher-MGB were used as described elsewhere [13, 15]. These primers target the conserved region of *L. infantum* of the kinetoplast DNA minicircles conserved region and amplify a fragment of 120 bp [16]. Parasite load was estimated using a standard DNA curve ranging from 1 ng to 0.1 fg of *L. infantum* (MHOM/BR/76/M4192) genomic DNA, as described elsewhere [15]. The standard curve and a negative control (no template DNA) were included in each PCR run, which were performed on an QuantStudio® 5 Real-Time PCR system (Applied Biosystems, Foster City, California, USA), in a final volume of 15 μl, containing 2 μl of DNA, 2.5 μl of nuclease-free water, 1.35 μl of each primer at 900 nM, 0.3 μl of TaqMan® probe at 200 nM and 7.5 μl of TaqMan® Fast Advanced Master Mix (Applied Biosystems, Foster City, California, USA). The cycling conditions were as follows: initial denaturation at 95 °C for 20 s, 40 cycles at 95 °C for 1 s and 60 °C for 20 s [15].

Data analysis was performed using QuantStudio® Design and Analysis Software v1.3.1 (Applied Biosystems, Foster City, California, USA). PCR-positive dogs were defined as cases and PCR-negative dogs as controls. The parasite load was categorized as described elsewhere [15], considering the estimated number of parasites per ml of blood as follows: low (0–10 parasites), medium (11–100 parasites), high (101–1000 parasites) or very high (>1000 parasites).

#### CBD1 gene and SNP analysis

Primers for *CBD1* (DEFEX1FW 5’-ATC CCT GCC CTA TAA ATA CCG-3’ and DEFEX1RW 5'-CCA AAC ACA GTC AGG GAT G-3') were designed using the Primer3Plus software [17], using *Canis lupus familiaris* genomic sequences deposited in the GenBank (NC_006598 for the *DEFB1* gene). The primers were designed to amplify the region between the promoter and intron 1 of the CBD1 gene with an expected amplicon size of 582 bp. The OligoAnalyzer software (http://www.idtdna.com/analyzer/applications/oligoanalyzer) was used to verify the quality of the selected primers based on the observation of the following criteria: melting temperature definition, primer dimer and hairpins formation. PCR standardization was performed in a final volume of 25 μl containing: 1.5 μl of each primer at a concentration of 10 pmol/μl; 7.5 μl of nuclease-free water, 12.5 μl of GoTaq Master Mix (Promega, Madison, Wisconsin, EUA) and 2 μl of DNA. The amplification profile was defined according to the melting temperature of the primer. A gradient was performed to find the perfect annealing temperature and the number of cycles was defined by the size of the fragment specific to the primer.

The amplification conditions for the *CBD1* gene were as follows: initial denaturation at 94 °C for 5 min, 35 cycles of (94 °C/30 s, 55 °C/30 s and 72 °C/30 s), and final extension at 72 °C for 10 min. The amplified products were analysed in 0.5% agarose gels, containing 1% of ethidium bromide and visualized with an ultraviolet transilluminator. All products were sequenced using same primers and the BigDye® Terminator v3.1 cycle sequencing kit (Applied Biosystems, Foster City, California, USA), in a Genetic Analyzer 3500 (Applied Biosystems, Foster City, California, USA).

Consensus sequences were generated based on the Phred 40 values using the Staden package [18] and validated by BLAST (http://blast.ncbi.nlm.nih.gov/). The sequences were aligned using the MEGA v.5.3 software [19], which was also used to visualize and quantify the polymorphisms.

### Data analysis

The inter-population differentiation (*F*_ST_) and the intra-population genetic variability (*F*_IS_) were assessed using Arlequin version 3.11 [20] and GenePop version 4.0 [21]. The correlation between genetic and geographic distances was assessed with Mantel test using Arlequin version 3.11 [20]. Logistic regression was performed to evaluate SNPs and haplotype associations with the infection (case-control) were assessed using SNPStats software (http://bioinfo.iconcologia.net/SNPstats_web), assuming a 5% significance level [22]. Statistical power was calculated using G*Power software [23]. This case-control study has > 80% power to identify a genotype association at the 5% significance level.

## Results

The real-time PCR positivity values for *L. infantum* were 31.6% (30/95) in São Raimundo Nonato and 36.8% (70/190) in Sobral. The canine population of Putignano showed a positivity of 33.3% (34/102). Regarding the parasitic load among the 134 positive dogs, one was considered very highly positive, three were highly positive, nine were medium positive and 121 were low positive (Additional file 1: Table S1).

The PCR product for the *CBD1* gene showed a band size of approximately 600 bp. The specificity of the PCR was confirmed by BLAST analysis, which presented high levels of similarity (99%) with a sequence of the *CBD1* gene (AC186962.12), which was used as a reference for designing the primers used in this study.

All dog samples were sequenced and DNA fragments of 582 bp were obtained. In the polymorphism analysis, 572 conserved sites and 10 variable sites were detected, and nine of them were parsimony informative. The informative sites were found in the 5’ untranslated region (position in the chromosome 16: 58881447) and in intron (position in the chromosome 16: 58881356, 58881297, 58881294, 58881277, 58881159, 58881122, 58881093, 58881081) (see Tables 1, 2 and 3). No polymorphic sites were detected in the coding region (exons 1 and 2).

**Table 1:** Association between genetic polymorphisms in β-defensin-1 and with *Leishmania infantum* in dogs from Sobral, Brazil

**SNP**	Variation ID	Position^a^	Alleles	HWE	Possible genotype	Total frequency (*n* = 190)	Frequency in cases (*n* = 70)	Frequency in controls (*n* = 120)	Odds ratio (95% CI)	Logistic regression (*P*-value)
SNP1	rs852527703	58881447	C/T	0.14	T/T	0.23	0.19	0.25	0.51 (0.22–1.15)	0.17
					C/T	0.44	0.40	0.47	0.59 (0.30–1.15)	
					C/C	0.33	0.41	0.28	1.00	
SNP2	rs852380685	58881356	C/T	0.14	T/T	0.23	0.19	0.25	0.51 (0.22–1.15)	0.17
					C/T	0.44	0.40	0.47	0.59 (0.30–1.15)	
					C/C	0.33	0.41	0.28	1.00	
SNP3	rs850814192	58881297	A/G	0.59	G/G	0.51	0.49	0.52	1.00	0.73
					A/G	0.42	0.43	0.42	1.11 (0.60–2.06)	
					A/A	0.07	0.09	0.06	1.59 (0.49–5.10)	
SNP4	rs852670798	58881294	A/C	0.77	C/C	0.30	0.31	0.29	1.00	0.87
					A/C	0.51	0.49	0.52	0.86 (0.44–1.69)	
					A/A	0.19	0.20	0.18	1.01 (0.43–2.38)	
SNP5	rs852439766	58881277	C/T	0.082	T/T	0.25	0.26	0.25	1.00	0.89
					C/T	0.56	0.54	0.57	0.92 (0.45–1.86)	
					C/C	0.18	0.20	0.18	1.11 (0.45–2.72)	
SNP6	rs850643698	58881159	A/G	0.77	G/G	0.19	0.20	0.18	1.01 (0.43–2.38)	0.87
					A/G	0.51	0.49	0.52	0.86 (0.44–1.69)	
					A/A	0.30	0.31	0.29	1.00	
SNP7	rs853079810	58881122	A/G	0.19	G/G	0.25	0.26	0.25	1.00	0.91
					A/G	0.55	0.53	0.56	0.92 (0.45–1.87)	
					A/A	0.20	0.21	0.19	1.09 (0.45–2.61)	
SNP8	rs851268228	58881093	C/T	1	T/T	0.08	0.10	0.07	1.57 (0.53–4.71)	0.72
					C/T	0.41	0.40	0.41	1.03 (0.55–1.92)	
					C/C	0.52	0.50	0.52	1.00	
SNP9	rs850702571	58881081	A/G	0.24	G/G	0.48	0.50	0.48	1.00	0.88
					A/G	0.39	0.37	0.41	0.86 (0.46–1.63)	
					A/A	0.12	0.13	0.12	1.05 (0.41–2.67)	

^a^Position in the reference sequence of canine chromosome 16 (accession number: NC_006598.3)

*Abbreviations*: *SNPs* single nucleotide polymorphisms, *HWE* Hardy-Weinberg equilibrium

**Table 2:** Association between genetic polymorphisms in β-defensin-1 and with *Leishmania infantum* in dogs from São Raimundo Nonato, Brazil

SNP	Variation ID	Position^a^	Alleles	HWE	Possible genotype	Total frequency (*n* = 95)	Frequency in cases (*n* = 18)	Frequency in controls (*n* = 77)	Odds ratio (95% CI)	Logistic regression (*P*-value)
SNP1	rs852527703	58881447	C/T	0.22	T/T	0.32	0.43	0.26	1.00	0.10
					C/T	0.43	0.43	0.43	0.61 (0.23–1.61)	
					C/C	0.25	0.13	0.31	0.26 (0.07–0.95)	
SNP2	rs852380685	58881356	C/T	0.22	T/T	0.32	0.43	0.26	1.00	0.10
					C/T	0.43	0.43	0.43	0.61 (0.23–1.61)	
					C/C	0.25	0.13	0.31	0.26 (0.07–0.95)	
SNP3	rs850814192	58881297	A/G	1	G/G	0.51	0.60	0.46	1.00	0.44
					A/G	0.42	0.33	0.46	0.56 (0.22–1.40)	
					A/A	0.07	0.07	0.08	0.67 (0.12–3.80)	
SNP4	rs852670798	58881294	A/C	1	C/C	0.48	0.63	0.42	1.00	0.10
					A/C	0.43	0.33	0.48	0.46 (0.18–1.15)	
					A/A	0.08	0.03	0.11	0.20 (0.02–1.79)	
SNP5	rs852439766	58881277	C/T	0.41	T/T	0.20	0.17	0.22	0.50 (0.14–1.84)	0.47
					C/T	0.55	0.50	0.57	0.57 (0.21–1.56)	
					C/C	0.25	0.33	0.22	1.00	
SNP6	rs850643698	58881159	A/G	1	G/G	0.08	0.03	0.11	0.20 (0.02–1.79)	0.10
					A/G	0.43	0.33	0.48	0.46 (0.18–1.15)	
					A/A	0.48	0.63	0.42	1.00	
SNP7	rs853079810	58881122	A/G	0.41	G/G	0.16	0.13	0.17	0.60 (0.15–2.34)	0.67
					A/G	0.54	0.50	0.55	0.68 (0.26–1.78)	
					A/A	0.31	0.37	0.28	1.00	
SNP8	rs851268228	58881093	C/T	0.82	T/T	0.09	0.07	0.11	0.48 (0.09–2.61)	0.53
					C/T	0.45	0.40	0.48	0.65 (0.26–1.62)	
					C/C	0.45	0.53	0.42	1.00	
SNP9	rs850702571	58881081	A/G	0.53	G/G	0.64	0.73	0.60	1.00	0.43
					A/G	0.31	0.23	0.34	0.56 (0.21–1.53)	
					A/A	0.05	0.03	0.06	0.44 (0.05–4.22)	

^a^Position in the reference sequence of canine chromosome 16 (accession number: NC_006598.3)

*Abbreviations*: *SNPs* single nucleotide polymorphisms, *HWE* Hardy-Weinberg equilibrium

**Table 3:** Association between genetic polymorphisms in β-defensin-1 and with *Leishmania infantum* in dogs from southern Italy

SNP	Variation ID	Position ^a^	Alleles	HWE	Possible genotype	Total frequency (*n* = 102)	Frequency in cases (*n* = 33)	Frequency in controls (*n* = 69)	Odds ratio (95% CI)	Logistic regression (*P*-value)
SNP1	rs852527703	58881447	C/T	0.16	T/T	0.25	0.30	0.22	0.39 (0.12–1.23)	0.18
					C/T	0.42	0.48	0.39	0.44 (0.16–1.23)	
					C/C	0.33	0.21	0.39	1.00	
SNP2	rs852380685	58881356	C/T	0.16	T/T	0.25	0.30	0.22	0.39 (0.12–1.23)	0.18
					C/T	0.42	0.48	0.39	0.44 (0.16–1.23)	
					C/C	0.33	0.21	0.39	1.00	
SNP3	rs850814192	58881297	A/G	0.68	G/G	0.39	0.55	0.32	1.00	0.04
					A/G	0.45	0.39	0.48	2.08 (0.85–5.08)	
					A/A	0.16	0.06	0.20	5.73 (1.15–28.57)	
SNP4	rs852670798	58881294	A/C	0.24	C/C	0.47	0.30	0.55	1.00	0.05
					A/C	0.47	0.64	0.39	0.34 (0.14–0.83)	
					A/A	0.06	0.06	0.06	0.53 (0.08–3.30)	
SNP5	rs852439766	58881277	C/T	0.83	T/T	0.14	0.18	0.12	0.30 (0.08–1.15)	0.06
					C/T	0.49	0.61	0.43	0.34 (0.13–0.92)	
					C/C	0.37	0.21	0.45	1.00	
SNP6	rs850643698	58881159	A/G	0.33	G/G	0.06	0.06	0.06	0.58 (0.09–3.59)	0.11
					A/G	0.46	0.61	0.39	0.39 (0.16–0.95)	
					A/A	0.48	0.33	0.55	1.00	
SNP7	rs853079810	58881122	A/G	0.67	G/G	0.14	0.18	0.12	0.30 (0.08–1.13)	0.04
					A/G	0.44	0.58	0.38	0.31 (0.12–0.82)	
					A/A	0.42	0.24	0.51	1.00	
SNP8	rs851268228	58881093	C/T	0.68	T/T	0.18	0.06	0.23	6.80 (1.36–33.88)	0.02
					C/T	0.46	0.42	0.48	2.00 (0.82–4.92)	
					C/C	0.36	0.52	0.29	1.00	
SNP9	rs850702571	58881081	A/G	1	G/G	0.62	0.45	0.70	1.00	0.07
					A/G	0.34	0.48	0.28	0.37 (0.15–0.90)	
					A/A	0.04	0.06	0.03	0.31 (0.04–2.41)	

^a^Position in the reference sequence of canine chromosome 16 (accession number: NC_006598.3)

*Abbreviations*: *SNPs* single nucleotide polymorphisms, *HWE* Hardy-Weinberg equilibrium

In the population genetics analyses, the *F*_IS_ for all SNPs in *CBD1* revealed a low inbreeding rate, with values near zero. The *F*_ST_ (0.016) indicated low genetic differentiation, demonstrating that the three populations studied are genetically very similar and can be evaluated as a single population. The analysis of allele, genotype and haplotype frequencies showed agreement with the Hardy-Weinberg equilibrium.

When analysing the three populations together, we observed that none of SNPs in *CBD1* and haplotypes were associated with the *L. infantum* infection in dogs. With regard to the populations of Sobral and São Raimundo Nonato, data analysis demonstrated that none of the SNPs in *CBD1* found was associated with the presence of *L. infantum* (Tables 1 and 2).

The genotypes A/A and T/T within the SNPs 3 and 8, respectively, were associated with the risk of *L. infantum* infection in dogs from Italy. On the other hand, the genotypes A/C and A/G within the SNPs 4 and 7, respectively, were associated with protection against *L. infantum* infection (Table 3).

As far as haplotype association is concerned, no haplotypes were found to be associated with *L. infantum* infection in the studied dog populations.

## Discussion

In this study, we evaluated the possible association between SNPs in the *CBD1* gene and *L. infantum* infection in dogs from north-eastern Brazil and southern Italy. We found an association between some genotypes within SNPs in the *CBD1* gene and *L. infantum* infection in dogs from Italy, indicating that these SNPs could be potential genetic markers for the study of *L. infantum* infection susceptibility/resistance in these animals. Yet, no association was found between SNPs in the *CBD1* gene and *L. infantum* infection in the dog populations from Brazil, which may suggest that further studies are needed to confirm the associations found in Italy. These differences could also be related to differences in parasite strains infecting dogs in each studied area. This hypothesis is unlikely because *L. infantum* zymodeme MON-1 is the predominant strain of infecting dogs in the Mediterranean basin and apparently also in South America [24–26]. Another issue that should be considered is population stratification. As all SNPs are potentially under selection pressure, they may not be reliable to assess population stratification. Therefore, the associations found herein could also be due to the underlying structure of the Italian population.

The association between the SNPs in the *CBD1* gene and *L. infantum* infection in dogs here described, along with previous studies demonstrating the association between other gene polymorphisms and leishmaniasis in humans [27] and dogs [7–9, 28], indicate that genetic factors may be involved in the susceptibility of *L. infantum* to infection and disease development. Studies conducted by Sanchez-Robert et al. [8, 28] reported an association between a haplotype (TAG-8-141) that includes a SNP in the promoter region and a microsatellite in intron 1, and two SNPs (A4549G in intron 6 and C4859T in exon 8) located in the *Slc11a1* gene and susceptibility to leishmaniasis in dogs. In another study, Quilez et al. [9] found associations between polymorphisms in several genes (e.g. *Il7R*, *Lifr*, *C6, C7* and *Csf1r* genes) and CanL.

The results of the SNPs association analysis herein presented indicates that *CBD1* gene may be involved in the immune response of dogs to *L. infantum* infection. However, future analysis of the expression levels of the *CBD1* gene in dogs infected with *L. infantum* will provide more information about the function of this gene. A previous study evaluated the expression of beta-defensins (i.e. CBD1, CBD103 and CBD108) in the canine respiratory tract and their antimicrobial activity against the intracellular bacterium *Bordetella bronchiseptica* [11]. All three investigated beta-defensins were detected in respiratory cells and were also readily expressed in skin samples. The study demonstrated that cells infected with canine parainfluenza virus had lower levels of CBD1 and CBD108, as well as that CBD103 presents antimicrobial activity against *B. bronchiseptica*. Another study showed increased expression levels of the *CBD1* gene in the skin of dogs with atopic dermatitis compared with healthy individuals [12]. Hypothetically, differences in the expression of the *CBD1* gene in the skin of *L. infantum* infected dogs could be associated with the presence/absence/severity of skin lesions commonly seen in dogs with CanL. Again, further research is needed to evaluate the functional effects (if any) of the SNPs found herein and, from a broader perspective, to assess if CBD1 or other beta-defensins display any antimicrobial activity against *L. infantum*.

## Conclusion

This study demonstrates for the first time a possible association between SNPs in the *CBD1* gene and *L. infantum* infection in dogs. A functional validation of this hypothesis, for example, to assess the level of expression of β-defensin-1 in the skin of dogs carrying these SNPs, would be required.

## Additional file

Additional file 1: Table S1. Frequency of *Leishmania infantum* parasite load categories in dogs from Sobral (SOB) and São Raimundo Nonato (SRN), Brazil, and Italy (ITA). (DOCX 17 kb)
