# Supplementary material for: Canine β-defensin-1 (CBD1) gene as a possible marker for Leishmania infantum infection in dogs
Source: Parasit Vectors. 2017 Apr 20;10:199. doi: 10.1186/s13071-017-2130-8 (PMC5399410; doi:10.1186/s13071-017-2130-8)
Supplement: Additional file 1: Table S1. — Frequency of Leishmania infantum parasite load categories in dogs from Sobral (SOB) and São Raimundo Nonato (SRN), Brazil, and Italy (ITA). (DOCX 17 kb) [file 13071_2017_2130_MOESM1_ESM.docx]

**Additional file 1**

**Table S1** Frequency of *Leishmania infantum* parasite load^a^ categories in dogs from Sobral (SOB) and São Raimundo Nonato (SRN), Brazil, and Italy (ITA)

| Parasite load | SOB  *n* (%) | SRN  *n* (%) | ITA  *n* (%) | All  *n* (%) |
| --- | --- | --- | --- | --- |
| Low positive | 65 (92.9) | 27 (90) | 29 (85.3) | 121 (90.3) |
| Medium positive | 3 (4.3) | 2 (6.7) | 4 (11.8) | 9 (6.7) |
| High positive | 1 (1.4) | 1 (3.3) | 1 (2.9) | 3 (2.2) |
| Very high positive | 1 (1.4) | 0 (0.0) | 0 (0.0) | 1 (0.8) |
| Total | 70 (100.0) | 30 (100.0) | 34 (100.0) | 134 (100.0) |

^a^Determined by real time PCR. For more details, see Methods and ref. [15]
